# How is leader-member exchange related to teachers’ instructional practices in Türkiye? The mediating role of collective teacher innovativeness and learning effectiveness

**DOI:** 10.3389/fpsyg.2026.1960677

**Published:** 2026-10-23

**Authors:** Yurdagül Doğuş

**Affiliations:** Faculty of Education, Kocaeli University, Kocaeli, Türkiye

**Keywords:** collective teacher innovativeness, instructional practices, leader-member exchange, learning effectiveness, teacher motivation

## Abstract

**Introduction:**

How leadership processes that support teacher motivation are related to effective instructional practices remains a relevant topic in educational research. However, how leader-member exchange in schools is related to instructional practices is not yet well understood. Furthermore, there remains uncertainty in the literature regarding how the relationship between leader-member interaction and teachers’ instructional practices shapes collective innovation and learning effectiveness. In this respect, the aim of the study is to examine the mediating role of perceived collective teacher innovativeness and learning effectiveness in the association between leader-member exchange and teachers’ instructional practices.

**Methods:**

The sample consisted of 1185 teachers recruited from all 12 NUTS-1 regions of Türkiye through proportional regional stratification with voluntary participation within schools. Moreover, the study was conducted using quantitative and cross-sectional design, the study data were collected with structured scales, and Structural Equation Modeling was used in the analyses.

**Results:**

The analysis showed positive associations among all four constructs. Leader-member exchange had a small direct association with instructional practices (β = 0.10) and larger associations with perceived collective innovativeness (β = 0.34) and learning effectiveness (β = 0.25); the two mediators together carried about 63% of the total association (total indirect β = 0.17), with the learning pathway (β = 0.10) somewhat larger than the collective pathway (β = 0.07). The model explained 28.8% of the variance in instructional practices. Because the design is cross-sectional and all measures were self-reported, these results describe associations consistent with the proposed model rather than causal effects.

**Discussion:**

They suggest that relational leadership is related to instructional practice mainly through teachers’ perceptions of collegial innovativeness and their beliefs about their own professional learning.

## 1 Introduction

Teachers’ in-class instructional practices (IP) are considered a determining factor in student success as well as the overall quality and sustainability of education systems (Darling-Hammond, 2000; Hattie and Yates, 2013). Effective IP not only increases academic achievement, but also improves students’ motivation to learn, their level of engagement in class, and their lifelong learning skills (Goddard et al., 2019). Therefore, in-school structures and relationships supporting teachers’ pedagogical practices are critical to the lasting effects of reforms (Bryk et al., 2010; Turan, 2026). However, recent studies show that explaining teachers’ IP levels solely through individual professional competencies is insufficient; social relationships within the school, professional learning culture, and leadership structures also significantly shape teacher behavior (Bellibaş et al., 2021a; Luyten and Bazo, 2019). In this context, although the educational administration and leadership literature emphasizes the impact of school leadership on IP (Bellibaş et al., 2021b; Özdemir, 2019), the mechanisms by which this impact occurs are still not sufficiently clear. Especially the leader-member exchange (LMX), which expresses the quality of the relationship between school principals and teachers, stands out as a powerful structure that can shed light on this question (Graen and Uhl-Bien, 1995). However, holistic models that test the direct and indirect effects of LMX on IP are limited.

Empirical studies show that school environments open to collective innovativeness and professional learning have positive effects on school improvement and student achievement (Blömeke et al., 2021). In this context, an answer is sought to the question of how teachers’ collective innovativeness development tendencies and their effective participation in learning processes are related to leader-member relations. Collective teacher innovativeness (CTI) includes teachers’ efforts to collaboratively develop new teaching strategies, produce creative solutions to pedagogical problems, and embrace change (Thurlings et al., 2015). Because CTI is measured through individual teachers’ reports of how innovative the teaching staff in their school is, it is treated in this study as an individual teacher’s perception of collective innovativeness, rather than as a school-level characteristic based on aggregated data. This distinction is important because the construct is measured at the individual level, which also limits how the findings can be interpreted. Learning effectiveness (LE) refers to teachers’ belief that they can engage in, sustain, and benefit from professional learning to improve their own practice (Liu et al., 2016). It is also measured at the individual teacher level. The structure of LMX, which includes the dimensions of trust and emotional commitment (Liden et al., 1997) can create a school culture that can increase teachers’ motivation for innovativeness and learning. Therefore, it can support both the CTI and the LE. Previous research has established the two ends of this chain more clearly than the relationship between them. One line of research has shown that instructional and distributed leadership are associated with teachers’ classroom practices (Bellibaş et al., 2021a,b) and has examined shared practice and LE as mediators of these relationships (Bellibaş et al., 2022). Thus, LE as a mediator is not new to this literature. Another line of research has examined factors associated with collective teacher innovativeness, including teacher autonomy, collaborative culture, and professional learning across 48 countries (Nguyen et al., 2021), distributed leadership through job satisfaction and professional collaboration (Buyukgoze et al., 2024), and school leadership using multilevel TALIS (Teaching and Learning International Survey) data (Hsieh et al., 2026). Research that specifically examines LMX as a predictor is more recent and remains limited. Adams et al. (2025) examined LMX together with openness to experience and teacher creativity, Kidwell (2021) focused on the challenges of developing high-quality LMX in virtual settings, and Evers et al. (2024) linked LMX to innovative work behavior through the satisfaction of basic psychological needs. However, previous research has not examined whether the relationship between LMX and teachers’ instructional practices operates through two distinct pathways at the same time: a social pathway through perceived collective innovativeness and a learning pathway through teachers’ learning effectiveness. It is also unclear whether one of these pathways is stronger than the other. This is the specific question addressed in the present study. Accordingly, the study examines the direct associations of LMX with teachers’ perceived collective innovativeness and LE, as well as their indirect associations with instructional practice. At the same time, these relationships should not be understood as strictly one-way. Teachers who are already innovative and effective learners may also receive more supportive exchanges from their principals. Such reciprocal or reverse relationships cannot be ruled out in a cross-sectional design.

In the study, a structural model including LMX, IP, CTI, and LE variables were tested with data collected from a total of 1185 teachers. The study contributes to the literature in three specific and deliberately modest ways: it models perceived collective innovativeness and LE as two simultaneous routes rather than examining either in isolation, it compares their relative magnitudes within a single model, and it does so in a highly centralized education system in which the room available to principals for relational, pedagogically oriented leadership is institutionally constrained. These are extensions of existing work rather than the establishment of a new theoretical framework, and the study is presented as such. In this sense, the study aims to examine the mediating role of collective teacher innovativeness and learning effectiveness in the association between leader-member exchange and teachers’ instructional practices and to seek answers to the following questions:

1. Is there a significant relationship between teachers’ leader-member exchange, perceived collective teacher innovativeness, learning effectiveness, and instructional practices?
2. Is leader-member exchange significantly associated with teachers’ perceived collective teacher innovativeness, learning effectiveness, and instructional practices?
3. Do teachers’ perceived collective teacher innovativeness and learning effectiveness have a mediating role in the association between leader-member exchange and on instructional practices?

### 1.1 Context of the study

Türkiye’s centralized and hierarchical education system limits teachers’ instructional practices, their relationships with leaders, and their levels of innovativeness, it restricts principal-teacher interaction and teacher autonomy (Kılınç et al., 2018). There is increasing evidence in literature that the centralized structure reduces the feasibility of innovative efforts and limits the capacity of educational leaders to develop effective practices (Bellibaş, 2023). Although the new curriculum, which adopted a constructivist approach to education [Ministry of National Education (MoNE, 2005)], envisaged teachers adopting more interaction-based and innovative educational practices, the anticipated transformation did not occur to the expected extent. Current curriculum regulations, such as the “Türkiye Century Education Model (Türkiye Yüzyılı Maarif Modeli),” aimed to encourage innovativeness and digital transformation (MoNE., 2025b). The 2023 Education Vision (MoNE, 2018) document also supported structural changes aimed at increasing teachers’ digital skills and strengthening the interaction of school leaders with teachers. Additionally, technological infrastructure projects and exemplary school practices fostered action learning environments that support teachers’ professional development (MoNE, 2010). These reform studies reveal that teachers’ innovative behaviors and professional learning motivation have gained significant importance in Türkiye. Rather than explaining the findings in terms of a uniform national culture, this study focuses on specific institutional features of the Türkiye education system. Curricula, textbooks, assessment arrangements, and much of in-service training are centrally determined. Principals work within a hierarchical administrative structure and have substantial bureaucratic responsibilities, while teachers have relatively limited formal autonomy over curricular decisions. Professional learning is also largely provided through standardized, centrally organized in-service training rather than school-based inquiry and collaboration (Bellibaş, 2023; Kılınç et al., 2018; MoNE., 2025b). Under these conditions, principals have limited formal control over classroom practices, making their relationships with teachers particularly relevant to understanding instructional practice. Cultural accounts that describe Türkiye as a collectivist society (Hofstede, 2011) are consistent with this perspective, but culture is treated as one possible context rather than as a direct explanation of the findings. National cultures are not uniform or deterministic, and considerable differences may exist across regions, school types, and individual schools within Türkiye. The study therefore does not claim that Turkish schools are fundamentally different from schools in other education systems. Instead, it examines teachers’ instructional practices, their relationships with school leaders, and the innovation- and learning-related pathways that may connect these factors within the specific institutional context of the Türkiye education system.

### 1.2 Theoretical foundations of the study

The theoretical basis of this study was formed within the framework of Social Exchange Theory (SET) and Organizational Learning Theory (OLT). SET suggests that individuals behave in social relationships based on the perception of reciprocity, trust, and fairness (Blau, 1964). When leaders provide social support and appreciation to employees, employees are more likely to commit to their jobs and contribute to the organization. LMX is grounded in this theoretical basis and explains how trust-based interactions between school leaders and teachers are related to teachers’ motivation, innovative behaviors, and attitudes toward learning. OLT emphasizes how individuals and teams achieve organizational learning through creating new knowledge, sharing and applying this knowledge (Argyris and Schön, 1996). According to OLT, teachers’ participation in professional development and reflecting what they have learned in the classroom are critical to improving the quality of education.

The two theories are used for different purposes in the model, with each explaining a specific part of the proposed relationships. SET explains why teachers may be willing to invest additional effort when they perceive their relationship with the principal as trustworthy, fair, and reciprocal. This may include contributing to and maintaining a climate of innovation among colleagues. Thus, SET explains the first part of the model, namely the relationships between LMX and the two mediators. However, SET does not explain how this effort may lead to changes in classroom practice. OLT addresses this part by explaining how teachers’ involvement in collegial activities and their own professional learning can contribute to changes in classroom practices through the creation, sharing, and application of pedagogical knowledge. OLT therefore explains the second part of the model, linking the two mediators to instructional practice. Neither theory alone provides a complete explanation of the proposed model: SET does not explain how knowledge is translated into practice, while OLT does not explain why teachers may become willing to engage in the learning process. For this reason, the two theories are combined to explain different but complementary parts of the model.

Within this framework, the two mediators represent two distinct pathways rather than two measures of the same process. The first pathway is social and collective. A supportive relationship with the principal is associated with teachers perceiving their colleagues as open to change. When teachers perceive their colleagues as innovative, they may benefit from shared ideas and examples of new practices, while also feeling more comfortable trying approaches that differ from established routines (Goddard et al., 2007; Thurlings et al., 2015). The second pathway is individual and learning-oriented. A supportive relationship with the principal is associated with teachers’ belief in their ability to engage in and sustain professional learning. This belief may support instructional practice by helping teachers acquire and apply new pedagogical knowledge (Liu et al., 2016; Supovitz et al., 2010). Thus, the first pathway concerns how teachers perceive their colleagues’ practices, whereas the second concerns how teachers perceive their own capacity for learning. These pathways are expected to operate in parallel because teachers may perceive their colleagues as highly innovative while having less confidence in their own learning, or they may have strong confidence in their own learning despite perceiving less innovation among colleagues. It is also important to note that the collective pathway is based on individual teachers’ perceptions of their colleagues. Therefore, the model examines a perceived collective process rather than a school-level process based on aggregated data. Taken together, SET helps explain the relationship between LMX and teachers’ perceptions and attitudes, while OLT helps explain how these factors are related to classroom instructional practices. The model presented in Figure 1 examines these two relational and learning-based pathways through which LMX may be associated with instructional practice. The model brings together two established theories and applies them to a combination of constructs that has received limited attention in a centralized education system; it is therefore not proposed as a new theory.

**FIGURE 1:**
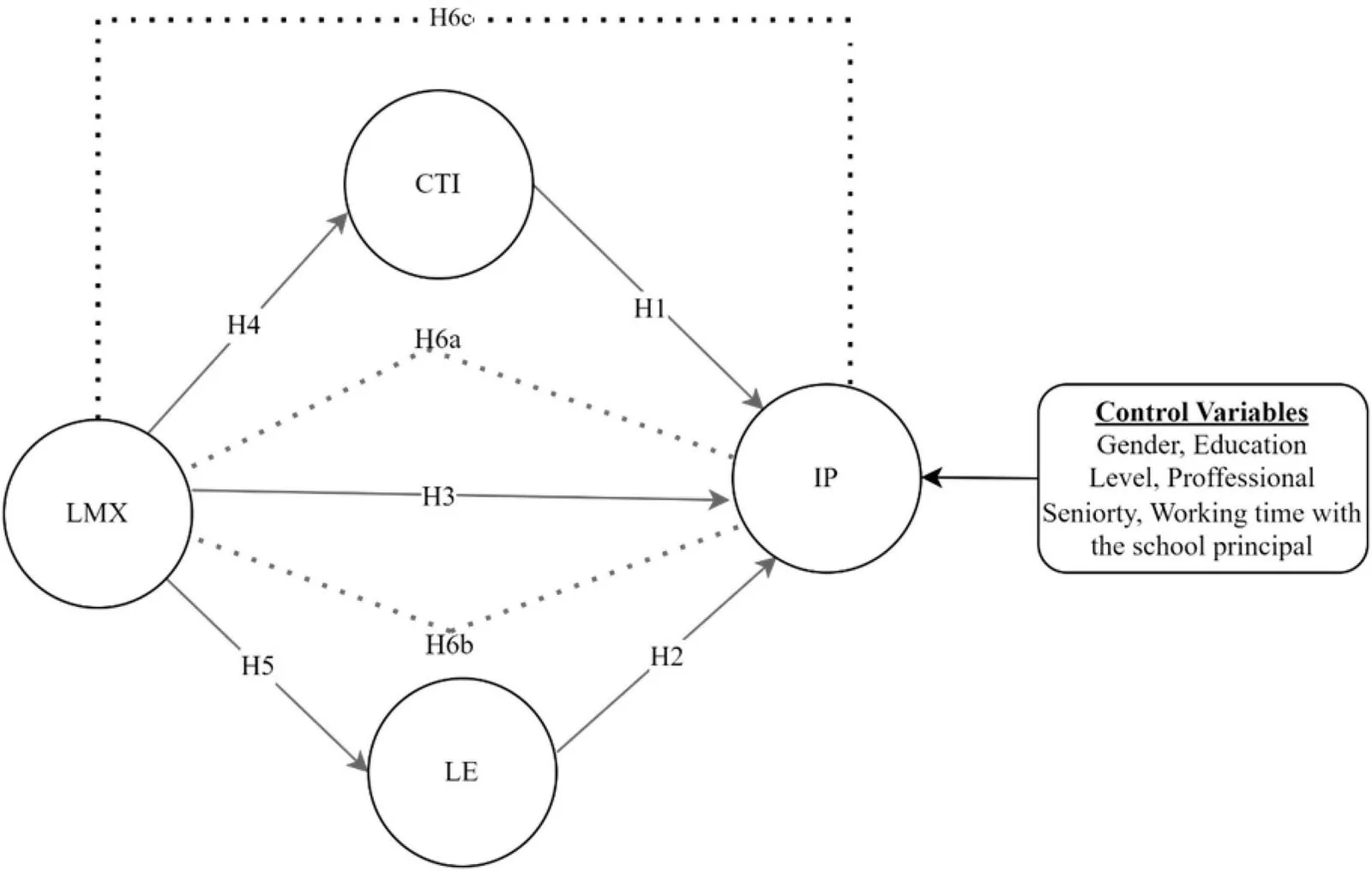
Conceptual model of the study.

#### 1.2.1 Instructional practices

In-class IP that directly affect student learning should be at the core of school improvement processes (Bellibaş et al., 2021b). Hattie (2012) stated that the strategies teachers use in classrooms are the most influential factor on student success, particularly formative assessment, clear presentation, effective feedback, small group work, metacognitive, collaborative, and problem-based learning methods create strong effects. Similarly, Marzano et al. (2001) found that feedback, self-regulation, meaningful assignments, hypothesis testing, goal setting, and cooperative learning are key elements supporting effective teaching. On the other hand, Lockheed and Komenan (1989) drew attention to structural components such as teaching materials, time management, evaluation, and feedback, while Stronge et al. (2007) highlighted teacher competencies such as adapting teaching to students’ needs, asking meaningful questions, and classroom management. This study is based on the instructional practice framework developed by Özdemir (2019), which includes practices such as increasing students’ interest in the course, ensuring active participation, and needs-based group studies.

#### 1.2.2 Collective teacher innovativeness

In the context of education, innovativeness aims to improve learning outcomes through innovation in areas such as pedagogical approaches, teaching methods, and organizational structures (Serdyukov, 2017). Innovativeness is defined at the individual level by teachers’ openness to new ideas and their willingness to implement these ideas (Buske, 2018), while at the collective level it is defined by the existence of a common climate for innovativeness and a collaborative culture among teachers (Schwabsky et al., 2019). In other words, collective teacher innovativeness (CTI) refers to the common attitudes and behaviors of a teacher group toward innovativeness. According to Buske (2018), CTI is not only the sum of individual innovativeness but also a collective and independent characteristic reflecting the group’s tendencies toward innovativeness. This study is based on the framework regarding teachers’ openness to change and their efforts to develop new ideas defined within TALIS 2018 (OECD [Organisation for Economic Co-operation and Development], 2019). A conceptual and methodological qualification is necessary at this point. Although CTI is defined in the literature as a group-level property, it is measured in the present study, as in TALIS itself, by asking individual teachers to report on the openness to change of most teachers in their school. Establishing CTI as a school-level construct would require aggregating these reports within schools and demonstrating within-group agreement and between-group reliability, which in turn requires school identifiers. Such identifiers were not collected in the present study, so aggregation and the associated agreement statistics could not be computed. CTI is therefore conceptualized, measured and interpreted throughout this article at the individual level, as a teacher’s perception of the collective innovativeness of the staff in their school. This is not a purely terminological point: the findings speak to how a teacher’s perception of a collectively innovative environment relates to that teacher’s own practice, and not to a demonstrated school-level process.

Today, it is becoming increasingly difficult for a single teacher to individually develop and implement instructional innovations, especially in the context of 21st century skills (Nguyen et al., 2021). Implementing innovative ideas is possible with teacher teams that have a collaborative vision and a culture of sharing (Doronin et al., 2020). An innovative school climate can provide environments that support instructional development by strengthening interaction, sharing, and collaboration among teachers (Thomson and Hillman, 2020). When teachers are given the opportunity to learn and practice effective teaching methods and use innovative materials, they are more likely to change and improve their IP (Parise and Spillane, 2010). The mechanism assumed in the hypothesis below is the one specified by organizational learning theory: where a teacher perceives colleagues as collectively open to change, pedagogical knowledge is more likely to be created, shared and applied, and the individual cost and risk of departing from an established routine is lower (Thurlings et al., 2015). The hypothesis therefore concerns a perceived collective climate in relation to individual practice, and not a demonstrated group-level effect. Accordingly, the first hypothesis of the study is as follows:

H1: Teachers’ perceived collective teacher innovativeness is positively and significantly associated with their instructional practices.

#### 1.2.3 Learning effectiveness

Learning effectiveness refers to teachers’ commitment to learning and their self-belief in this process in order to improve their instructional practice (Liu et al., 2016). Since teacher self-efficacy is related to many closely related concepts, such as participation in general professional learning activities and motivation to learn, its boundaries need to be clearly defined. For example, teacher self-efficacy concerns beliefs about implementing action plans in a defined area, and the instructional domain of teacher self-efficacy reflects beliefs regarding instruction, classroom management, and student participation (Bandura, 1977). LE, on the other hand, concerns a teacher’s beliefs about their own learning rather than the instructional activities they undertake. LE is also not related to the frequency of teachers’ participation in professional learning activities. In other words, it does not refer to the actual number of professional learning activities in which a teacher participates or to workplace learning among colleagues that has concretely taken place. LE refers to teachers’ efforts to continue learning under various conditions (Liu et al., 2016). Furthermore, LE is not the same as learning motivation, which is related to the pleasure, interest, and satisfaction derived from learning (Deci et al., 1991). LE is teachers’ belief that they will continue to learn even under very challenging conditions (Liu et al., 2016).

This study adopts the conceptualization of LE established by Liu et al. (2016). Accordingly, LE reflects teachers’ beliefs regarding their motivation, abilities, and perseverance as learners (Liu et al., 2016). In other words, LE is related to teachers’ effective learning and indicates that a teacher will continue to strive to improve their learning even when they do not feel well, are overwhelmed, are not supported by skeptical colleagues, or faces financial and administrative problems stemming from the system, a teacher will still strive to improve their learning. It also describes a teacher’s belief that if they work hard enough, they will continue to develop their teaching skills, and that they can learn more effectively when they receive help from colleagues (Bellibaş et al., 2019). LE reflects a teacher’s assessment of their own capacity to sustain and ensure the continuity of their learning process and to benefit from it, as well as their self-directed belief regarding their own learning process (Liu et al., 2016). To measure LE in this study, the “Learning Effectiveness” subscale of the “Teacher Agency Scale” developed by Liu et al. (2016) was used. Both the scale’s developers and Bellibaş et al. (2019), who adapted it into Turkish, referred to the concept as “learning effectiveness.” Therefore, the term “learning effectiveness” has been retained in this study as well.

The theoretical reason for expecting LE to mediate the association between LMX and instructional practice follows from the division of labor between the two frameworks described above. From the standpoint of social exchange theory, a teacher who experiences the relationship with the principal as trusting and reciprocal is more willing to invest discretionary effort in an activity whose returns are uncertain and deferred, and sustained engagement in one’s own professional learning is such an activity; the exchange relationship should therefore be associated with the teacher’s confidence that this learning can be undertaken and sustained. From the standpoint of organizational learning theory, it is this confidence, rather than the exchange relationship itself, that is proximal to classroom change, because new pedagogical knowledge has to be acquired, tried and adjusted by the teacher before it can alter routine practice (Argyris and Schön, 1996). LE is thus positioned in the model as the individual-level link between a relational condition and a behavioral outcome, which is why it is specified as a mediator rather than as an independent predictor.

A strong sense of LE is associated with teachers’ continued engagement in professional development to contribute to student success (Frost, 2006). It is emphasized in literature that teachers who participate in continuous and school-based professional learning activities tend to improve the quality of their teaching (Supovitz et al., 2010). A positive relationship was also found in a study conducted by Bellibaş et al. (2022) in the Türkiye sample, in which LE mediated the association between instructional leadership and teacher practices; the mediator is therefore not new to this literature, and what the present study adds is its examination alongside perceived collective innovativeness under a relational rather than instructional conception of leadership. Based on these theoretical and empirical findings, the hypothesis that teachers’ learning effectiveness is positively associated with IP is presented below:

H2: Teachers’ learning effectiveness is positively and significantly associated with their instructional practices.

#### 1.2.4 Leader-member exchange

Leader-member exchange refers to the quality of relational exchange between leader and employee (Liden, 1998). High-quality LMX enables leaders to empower teachers, increase participation, and share knowledge; and enables teachers to demonstrate loyalty, responsibility, and voluntary contributions (Krishnan, 2004). LMX consists of four key dimensions: affect, contribution, loyalty, and professional respect. The affect dimension refers to the emotional bond, mutual trust, and interpersonal attraction between the leader and the employee. Contribution is the perceived quantity and quality of functional activities carried out by both parties to achieve common goals (Dienesch and Liden, 1986). Loyalty reflects the level of commitment of employees to the leader and the organization, especially their willingness to support the leader in the face of challenges (Liden et al., 1997). Professional respect refers to the mutual respect that the leader and follower have for each other’s professional competence and prestige within the organization (Ionescu and Iliescu, 2021).

In the school context, high-quality relationships between principals and teachers can increase teachers’ motivation to improve and transform their IP. High-quality LMX relationships contribute to teachers’ feeling valued, increasing their perception of administrative support and enabling them to participate more effectively in school decision-making processes (Graen and Uhl-Bien, 1995). This can increase teachers’ motivation not only to perform their duties but also to continually review and improve their classroom IP and adopt innovative approaches. Because neither SET nor OLT alone predicts a direct relationship between LMX and instructional practice, H3 examines whether a direct association remains after accounting for the two theoretically proposed pathways. Including this direct path allows us to test the mediated relationships examined in H6a and H6b without assuming that the mediation is complete. In this context, the following hypothesis is proposed:

H3: Leader-member exchange is positively and significantly associated with teachers’ instructional practices.

Today’s schools need structural and relational environments that support teachers to undertake innovative behaviors. Studies conducted on different types of leadership in literature show that these types of leadership styles encourage collective innovativeness in teachers (Buyukgoze et al., 2024; Kılınç et al., 2024). Moreover, it was revealed that LMX is significantly associated with teachers’ change-oriented attitudes such as readiness for change (Thompson, 2018), participation in change (van Dam et al., 2021), and openness to organizational innovation (Bilge and Konakli, 2024). The mechanism assumed in this study derives from social exchange theory. A teacher who experiences the exchange with the principal as fair and reciprocal is more willing to invest discretionary effort in shared, collegial activity, and is correspondingly more likely both to contribute to and to perceive a staff that is collectively open to change (Graen and Uhl-Bien, 1995). Based on these findings, it is assumed that the quality relationships that school principals establish with teachers can strengthen collective innovativeness and LE, and the fourth hypothesis is as follows:

H4: Leader-member exchange is positively and significantly associated with teachers’ perceived collective teacher innovativeness.

High-quality LMX relationships facilitate teachers’ taking professional responsibility, acting more autonomously and effectively toward learning, creating a psychological and structural climate that supports their active participation in professional development processes (Graen and Uhl-Bien, 1995). This may encourage teachers to be active participants who take responsibility for the learning process, not just receivers of information. This interactive structure helps teachers demonstrate a higher level of responsibility and initiative in acquiring new knowledge and skills, continuously improving their pedagogical approaches, and making more informed decisions within the classroom (Vanblaere and Devos, 2016). Therefore, the following hypothesis was proposed to demonstrate that LMX provides a structure that enables teachers to be more autonomous, motivated, and effective in their professional learning processes:

H5: Leader-member exchange is positively and significantly associated with teachers’ learning effectiveness.

Empirical studies show that positive relationships that school leaders have with teachers create a climate that supports innovativeness in the school and contributes to the transformation of pedagogical practices by strengthening teachers’ sense of effectiveness in learning processes (Bellibaş et al., 2022). In this context, it can be argued that the association between LMX and IP is partly indirect, running through teachers’ perceived collective innovativeness and LE. High-quality LMX may be associated with teachers’ ability to generate and implement new ideas by collaboratively sharing their professional knowledge and creativity; thus, it may also be associated with teachers’ motivation to improve their in-class practices. Similarly, quality LMX may be associated with better IP through teachers’ learning effectiveness, that is, through their belief that they can direct and sustain their own professional learning. Since these paths represent conceptually distinct mechanisms, the specific indirect effect through each mediator was estimated separately and tested with bootstrapping, following recommendations for multiple mediation models (Hayes, 2022; Preacher and Hayes, 2008). Two specific mediation hypotheses were therefore formulated. A third statement, H6c, concerns the total indirect association carried by the two mediators together. Because the mediators are specified in parallel, with no path between them, this total is simply the sum of the two specific indirect effects; H6c therefore does not propose a separate mechanism and is not a test of anything beyond H6a and H6b. It is retained because the overall indirect association, and its share of the total association, is a quantity of interest in its own right when the two routes are compared.

H6a: Perceived collective teacher innovativeness mediates the association between leader-member exchange and teachers’ instructional practices.

H6b: Learning effectiveness mediates the association between leader-member exchange and teachers’ instructional practices.

H6c: Taken together, perceived collective teacher innovativeness and learning effectiveness carry a positive and significant total indirect association between leader-member exchange and teachers’ instructional practices.

## 2 Materials and methods

This study was conducted within a cross-sectional design based on quantitative methods. Cross-sectional studies aim to produce results by collecting data on the target population at a specific time (Cohen et al., 2013). Cross-sectional design was preferred in the study to measure and analyze the characteristics of the target population at a specific point in time.

### 2.1 Participants and data collection process

The universe of this study consists of 1,168,896 teachers working throughout Türkiye (MoNE, 2024). The geographical scope of the study was defined based on the Level 1 Statistical Region Classification (NUTS-1) determined by the Türkiye Statistical Institute (TUIK). In this context, Türkiye is divided into 12 main regions indicated by the codes TR1-TRC. The sampling frame of the study was created to cover all of these Level 1 regions. In this context, a stratified sampling method was adopted to represent the regional, demographic, and professional diversity of the teacher population as comprehensively as possible. However, due to the inability to access an individual-level sampling frame covering all teachers in Türkiye, it was not possible to construct a complete sampling frame that adhered to the Equal Probability of Selection Method (EPSEM) principle that guarantees an equal probability of selection for every individual. Consequently, NUTS-1 regions were treated as strata in the study; schools were selected randomly within each stratum, and teachers at the selected schools were invited to participate in the study on a voluntary basis. Each Level 1 region (e.g., TR1: Istanbul, TR2: Western Marmara) was treated as a separate stratum, and the number of invitations allocated to each stratum was set in proportion to that region’s share of the teacher population reported in the MoNE (2024) statistics. It should be stressed that this procedure secures proportional regional coverage but not a probability sample. Participation within the selected schools was voluntary, and the selection probability of any individual teacher is unknown; the sample is therefore best described as a regionally stratified volunteer sample. Proportional coverage of the regions does not by itself establish that the sample is representative of all teachers in Türkiye, and the findings are interpreted with this qualification in mind. The scales of the study were delivered to the participants via Google Forms and e-mail. Various measures were taken during the online data collection process to improve data quality and monitor the distribution of responses. In this context, all items were designed to be mandatory to prevent missing data, and response completion times were analyzed; forms completed in unusually short periods were evaluated for careless responses. In addition, the number of responses received from each NUTS-1 layer was regularly monitored throughout the data collection process, compared against established proportional targets, and additional reminder invitations were sent to layers with low representation levels. Participation in the study was entirely voluntary. Online informed consent was obtained from all participants, and participant confidentiality and data security were meticulously protected in line with ethical rules. Moreover, the sample size was calculated based on the Cochran formula, taking into account a 95% confidence level and a 5% margin of error, and a minimum of 384 participants was found to be sufficient. However, in order to increase the statistical power of the study, the sample was expanded and a total of 1500 teachers were invited to participate in the study. As a result of the approximately 4-months data collection process, 1203 teachers responded, a teacher-level response rate of 80.2%. Eighteen cases were subsequently excluded as multivariate outliers on the basis of the criterion described in the section “2.3 Data analysis,” leaving 1185 teachers (79.0% of those invited) in the analysis. School identifiers were not recorded during data collection, so the number of participating schools and school-level response rates cannot be reported; the response rate given here refers to teachers only. The distribution of the final sample across the twelve NUTS-1 regions is shown in Table 1. The achieved regional distribution tracked the proportional targets closely. When the sample was compared with the regional distribution of the 1.187,409 teachers in formal education reported in the most recent national statistics (MoNE., 2025a), the share of each region in the analyzed sample differed from its share of the teacher population by 0.4 percentage points or less. The largest deviation was for Eastern Marmara (9.5% of the sample against 9.9% of the teacher population), and the two distributions did not differ significantly [χ^2^(11) = 0.71, *p* > 0.99; Table 1]. Of the 1185 teachers who participated in the study, 929 (78.4%) were female and 256 (21.6%) were male. Of the participants, 76 (6.4%) worked in preschools, 278 (23.5%) in primary schools, 387 (32.7%) in secondary schools, 343 (28.9%) in high schools, and 101 (8.5%) in other institutions. A total of 773 teachers (65.2%) had a bachelor’s degree, and 412 teachers (34.8%) had a postgraduate degree. The average length of service of teachers was calculated as 14.15 years (SD = 8.05), and the average length of service with a school principal was calculated as 2.93 years (SD = 2.50).

**TABLE 1:** Distribution of the analyzed sample across NUTS-1 regions (*n* = 1185) and share of the national teacher population.

NUTS-1 code	Region	*n*	%	Teacher population %^a^
TR1	Istanbul	175	14.8	14.9
TR2	Western Marmara	47	4.0	3.9
TR3	Aegean	143	12.1	12.3
TR4	Eastern Marmara	113	9.5	9.9
TR5	Western Anatolia	121	10.2	10.3
TR6	Mediterranean	165	13.9	14.1
TR7	Central Anatolia	63	5.3	5.2
TR8	Western Black Sea	66	5.6	5.4
TR9	Eastern Black Sea	40	3.4	3.2
TRA	Northeastern Anatolia	35	3.0	2.9
TRB	Central Eastern Anatolia	66	5.6	5.2
TRC	Southeastern Anatolia	151	12.7	12.7
Total		1185	100	100

*^a^*Share of each region in the 1,187,409 teachers working in formal education in the 2024/’25 school year (MoNE., 2025a,Table 1.17). Goodness of fit of the sample to these shares: χ^2^(11) = 0.71, *p* > 0.99.

Although participants work at different school levels (preschool, elementary school, middle school, and high school), the constructs examined in this study (LMX, CTI, LE, and IP) have been conceptualized as general teacher characteristics independent of school level, and the measurement instruments used were developed for teachers at all school levels and tested for validity and reliability. For example, the TALIS 2018 framework on which the CTI measurement is based brings together teachers from different levels within a single analytical framework (OECD [Organisation for Economic Co-operation and Development], 2019). Therefore, combining these groups into a single analysis is consistent with previous large-scale studies in the field. However, the possibility that respondent characteristics may vary by level should be taken into account, and it is recommended that future research formally test measurement invariance across school levels.

### 2.2 Variables and data collection tools

Independent (predictor) variable: leader-member exchange scale (LMES). LMES, developed by Liden (1998), was adapted into Turkish by Öztürk (2015). The scale’s affect (sample item: working with my school principal is very fun), contribution (sample item: if I make a forgivable mistake, my principal will defend me to other staff), loyalty (sample item: I do not shy away from doing the most difficult jobs for my school principal), and professional respect (sample item: I appreciate my school principal’s professional skills) dimensions consist of three items each. The items of the scale are answered on a 5-point Likert scale (1 = I completely disagree, 5 = I completely agree). The results of the Confirmatory Factor Analysis (CFA) applied for this study confirmed the four-dimensional structure of the scale (χ^2^/df = 2.86; RMSEA = 0.040; CFI = 0.99; TLI = 0.99; SRMR = 0.015). The Cronbach’s alpha (α) coefficients for the scale were found to be 0.94 for the emotion dimension, 0.92 for the contribution dimension, 0.87 for the loyalty dimension, and 0.95 for the professional respect dimension. The composite reliability (CR) values were calculated as 0.94, 0.92, 0.87, and 0.94, respectively. The Average Variance Extracted (AVE) values for the emotion, contribution, loyalty, and professional respect dimensions were determined to be 0.85, 0.80, 0.70, and 0.86, respectively.

Mediator variable-I: collective teacher innovativeness scale (CTIS). CTIS was developed to measure teachers’ level of innovativeness development as a team. It is a 5-point Likert-type scale (1 = Completely disagree, 5 = Completely agree) with 4 items used in the 2018 TALIS (Teaching and Learning International Survey) (sample item: most teachers in this school are open to change) (OECD [Organisation for Economic Co-operation and Development], 2019). The scale was adapted into Turkish by Demir (2024). CFA fit values were determined to be within appropriate ranges for the study (χ^2^/df = 2.27; RMSEA = 0.033; CFI = 1.00; TLI = 0.99; SRMR = 0.003). Additionally, it was observed that the Cronbach alpha (α), CR, and AVE values of the scale were 0.92, 0.94, 0.77, respectively.

Mediator variable-II: Learning Effectiveness Subscale (LES). The six-item “Learning Effectiveness Subscale” of the Teacher Agency Scale, developed by Liu et al. (2016) in order to determine the learning effectiveness levels of teachers and adapted to Turkish by Bellibaş et al. (2019) was used. LES is a 5-point Likert-type scale (1 = I completely disagree, 5 = I completely agree) (sample item: even when I feel bad, I can actively participate in professional learning activities). In this study, all fit indices of CFA met the recommended ranges (χ^2^/df = 1.93; RMSEA = 0.028; CFI = 0.99; TLI = 0.99; SRMR = 0.014). Cronbach alpha (α), CR, and AVE values of LES were found to be 0.75, 0.80, and 0.41, respectively.

Dependent variable: Instructional Practices Scale (IPS). IPS is a one-dimensional scale with 7 items (sample item: I can attract students’ attention to the lesson) developed by Özdemir (2019) to determine teachers’ in-class instructional practices. The scale is a 5-point Likert-type scale on which participants rate each item from 1 (I completely disagree) to 5 (I completely agree). For the study, all fit indices for CFA were found to be within appropriate ranges (χ^2^/df = 2.95; RMSEA = 0.041; CFI = 0.99; TLI = 0.98; SRMR = 0.017). Additionally, according to the study results, the Cronbach alpha (α) of the scale was calculated as 0.83, CR 0.85, and AVE 0.46. The reliability and validity coefficients reported in this section were obtained from a separate CFA of each instrument. Those reported in the section “3 Findings” come from the full measurement model, in which all latent variables were estimated simultaneously; the small differences between the two sets of values (for example, AVE = 0.41 versus 0.42 for LE, and 0.46 versus 0.48 for IP) arise from this difference in model specification. All validity judgments in this article are based on the full measurement model.

Control variables: previous studies have shown that gender may be associated with teachers’ instructional behaviors and self-efficacy beliefs; that educational level reflects differences in pedagogical knowledge and competencies; and that professional seniority represents cumulative professional experience that supports instructional quality (Asare, 2021; Dada, 2015). In addition, the length of time working with the school principal is considered one of the key determinants of the quality of leader-member interaction (Liden, 1998). Accordingly, gender, educational level, professional seniority, and the length of time working with the school principal were included in the model as control variables. The aim was thus to evaluate the unique contributions of the primary predictor variables in explaining instructional practices, free from the influence of other potential factors.

### 2.3 Data analysis

Analysis of the study data was carried out using Mplus 8.3 statistical software. In the study, the relationships between the variables and possible mediation effects were tested using the maximum likelihood estimation method within the scope of multivariate structural equation modeling (SEM). In data analysis, Covariance-Based Structural Equation Modeling (CB-SEM) was preferred over Variance-Based Structural Equation Modeling (VB-SEM/PLS-SEM). This preference is based on four main reasons. First, the aim of the study is to test the model developed on theoretical grounds using a confirmatory approach, rather than to conduct exploratory or predictive analysis. Second, all measurement instruments used in the study consist of scales whose validity and reliability have been established in the literature. Third, the sample size (*n* = 1,185) is sufficient to allow for the reliable and stable application of the maximum likelihood estimation method. Finally, the data satisfy the condition of multivariate normality, which is one of the fundamental assumptions of CB-SEM. Considering these characteristics, it was determined that CB-SEM is the most appropriate analytical approach in terms of the study’s objective and data structure. In contrast, VB-SEM/PLS-SEM is recommended for studies conducted with small sample sizes, where the assumption of multivariate normality is not met, and where the focus is more on exploration and prediction rather than theory testing (Hair et al., 2017; Kline, 2015). Before modeling, the distributional properties of the data were examined in two steps that should be kept distinct. First, univariate skewness and kurtosis were computed for the composite variables at the sample level; these are descriptive statistics of the distributions and were not used to delete cases. Second, multivariate outliers were identified case by case using the Mahalanobis distance (D^2^) computed on the four composite scores (LMX, CTI, LE and IP), evaluated against the χ^2^ distribution with df = 4 and a critical value of 18.47 at *p* < 0.001 (Tabachnick and Fidell, 2013). Eighteen cases (1.5% of the 1203 responses) exceeded this value and were excluded; no case was removed on any other basis. After exclusion, skewness ranged from −0.599 to −0.131 and kurtosis from −0.525 to 0.588, within the ±1.5 range. The data file retained for analysis contains only the 1185 cases that remained after this step; the 18 excluded responses were not kept, so the model could not be re-estimated with them included. As an alternative check on whether the results depend on where the exclusion threshold was set, the structural model was re-estimated after applying two stricter Mahalanobis criteria to the retained cases (Aguinis et al., 2013): the critical value at *p* < 0.01 (13.28), which removed a further 23 cases (*n* = 1162), and the critical value at *p* < 0.05 (9.49), which removed a further 87 cases (*n* = 1098). These re-estimations were carried out in semopy (Igolkina and Meshcheryakov, 2020) with the same specification, which reproduced the coefficients reported in the section “3 Findings” to within 0.01. Under the *p* < 0.01 criterion, model fit was unchanged (RMSEA = 0.042; CFI = 0.96; TLI = 0.95) and each structural coefficient changed by 0.04 or less (LMX → CTI = 0.38, LMX → LE = 0.28, LMX → IP = 0.07, CTI → IP = 0.22, LE → IP = 0.43). All paths remained significant, and the 95% bootstrap confidence intervals (5000 resamples) of the specific indirect effects through perceived collective innovativeness (β = 0.08, 95% CI [0.056, 0.111]) and LE (β = 0.12, 95% CI [0.086, 0.161]) did not include zero. The *p* < 0.05 criterion gave the same pattern; the largest change was for LE → IP (0.47). Removing further cases from the upper tail of the distribution therefore did not alter any conclusion. This check cannot show how the 18 excluded cases themselves would have affected the estimates, and this is acknowledged as a limitation. Additionally, the distribution of points observed in Q-Q graphs close to the 45-degree reference line and the appearance of an ellipse form in multivariate scatter diagrams indicate that the data are suitable for multivariate normality. In addition, the assumption of multivariate normality was formally tested based on Mardia (1970) multivariate skewness and kurtosis coefficients. The results indicate that the data satisfy the assumption of multivariate normality and possess the distribution properties required for covariance-based structural equation modeling. Multicollinearity among the predictors was not a concern: the correlations among LMX, CTI and LE were all below 0.35 (Table 2), and the largest variance inflation factor among the predictors of IP was 1.16 (Field, 2024). Diagnostics designed for single-equation regression, such as the Durbin–Watson statistic, condition indices and tolerance values, are not informative for a cross-sectional latent-variable model and are therefore not reported. Discriminant validity among the latent constructs, which is the relevant question in SEM, was assessed separately with the Fornell–Larcker criterion and the HTMT ratio, as reported in the section “3 Findings.”

**TABLE 2:** Descriptive statistics of the study and Pearson correlation analysis results (*n* = 1185).

Variables	M	SD	1	2	3	4	5	6	7	8
1. LMX	3.35	0.92	–	0.307^**^	0.262^**^	0.242^**^	0.020	0.043	0.075^**^	0.040
2. CTI	3.45	0.90		–	0.214^**^	0.293^**^	0.022	0.010	−0.032	−0.023
3. LE	4.01	0.54			–	0.430^**^	−0.020	0.039	−0.110^**^	−0.008
4. IP	3.83	0.60				–	−0.068^*^	0.069^*^	−0.085^**^	−0.004
5. Professional seniority	14.15	8.05					–	0.155^**^	0.154^**^	0.077^**^
6. Working time with the school principal	2.93	2.50						–	0.014	0.024
7. Gender	–	–							–	0.069^*^
8. Education level	–	–								–

*p < 0.05;

^**^p < 0.01, M, mean; SD, standard deviation; LMX, leader-member exchange; CTI, collective teacher innovativeness; LE, learning effectiveness; IP, instructional practices; Gender, the reference group is female; Education Level, the reference group is undergraduate.

The structural model was specified as follows. LMX was modeled as a second-order latent variable indicated by its four first-order dimensions (affect, contribution, loyalty and professional respect), and CTI, LE and IP were modeled as first-order latent variables indicated by their items. LMX was specified as a predictor of CTI, LE and IP, and CTI and LE as predictors of IP. The four control variables (gender, educational level, professional seniority and length of service with the current principal) were included as predictors of IP only and were not specified as predictors of the mediators. All parameters were estimated with maximum likelihood. Because all items were mandatory in the online form, there were no missing data. The specific indirect effects through CTI and LE and their sum were estimated with 5000 bootstrap resamples and evaluated with 95% bootstrap confidence intervals (Hayes, 2022).

Teachers are nested within schools, and teachers in the same school share a principal, an organizational climate and a professional learning context. Because school identifiers were not recorded during online data collection, this clustering could not be modeled, and the analyses treat observations as independent. If responses are correlated within schools, standard errors may be underestimated and the precision of the parameter estimates overstated (Hox et al., 2018). The only grouping variable available in the data set is the NUTS-1 region. As a partial check, intraclass correlation coefficients (ICC[1]; Bliese, 2000) were computed for the four composite scores across the twelve regions; they ranged from 0.00 (CTI) to 0.015 (LE), indicating that regional clustering is negligible. This check does not speak to clustering at the school level, where intraclass correlations for constructs such as LMX and perceived collective innovativeness could be considerably higher, and the results should be read with this unmodeled dependence in mind.

All constructs were measured with self-report scales completed by the same respondents at a single point in time, so common method variance and common-source inflation of the correlations are a genuine risk (Podsakoff et al., 2003, 2012). Two kinds of information bear on this risk and are kept separate here. First, several procedural steps were taken at the design stage: respondents were assured of confidentiality and anonymity, established scales with documented validity were used, the four instruments refer to different referents (the principal, colleagues, the respondent’s own learning and the respondent’s own classroom practice), and completion times were monitored so that careless responses could be identified. Second, statistical diagnostics were examined after data collection. Harman’s single-factor test (Harman, 1967) indicated that a single factor extracted from all items accounted for 32.19% of the variance. The full-collinearity VIF values proposed by Kock (2015), calculated from the construct-level correlation matrix, were 1.166 (LMX), 1.174 (CTI), 1.272 (LE) and 1.305 (IP) (all below section “3.3 Structural model”). In addition, a one-factor measurement model in which all 29 items loaded on a single latent variable fitted the data very poorly in comparison with the hypothesized model (Table 3). None of these diagnostics is a sensitive test of method variance. Harman’s test has low power and cannot show that method variance is absent (Fuller et al., 2016; Podsakoff et al., 2003), and the full-collinearity approach was developed for variance-based rather than covariance-based SEM, so it is reported here as supplementary rather than decisive evidence. An unmeasured latent method construct (Williams et al., 2010) was also specified, with every item loading on an orthogonal method factor in addition to its substantive factor; because the four LMX dimensions share a strong general factor, however, the method factor could not be separated from the higher-order LMX factor and the solution was not interpretable, so this approach is not reported as evidence. Taken together, the diagnostics make it unlikely that method variance is the sole source of the observed associations, but they cannot exclude some inflation of the coefficients. Common-source bias is therefore treated as a limitation of the study rather than as a problem that has been ruled out. Before performing the main analyses of the study, descriptive statistics such as mean (M), standard deviation (SD) were used to reveal the general structure of the data and Pearson correlation analysis (r) was used to determine the relationships between the variables. The hypotheses of the study were tested with SEM analysis. In this context, while evaluating the goodness of fit of the measurement model, fit indices such as chi-square/degree of freedom (χ^2^/df) ratio, Root Mean Square Error of Approximation (RMSEA), Standardized Root Mean Square Residual (SRMR), Tucker-Lewis Index (TLI), Comparative Fit Index (CFI), Incremental Fit Index (IFI), Goodness of Fit Index (GFI) and Adjusted Goodness of Fit Index (AGFI) were used. A χ^2^/df value below 5, RMSEA and SRMR values of 0.08 or below, and CFI, TLI, IFI, GFI and AGFI values above 0.90 were taken to indicate an acceptable level of fit, with CFI and TLI values at or above 0.95 indicating good fit (Hu and Bentler, 1999; Schermelleh-Engel et al., 2003). During the analysis process, the compatibility of the latent and observed variables in the model was ensured, and then the direct and indirect effects between the variables were examined with SEM. In order to test the significance of the results obtained, bootstrap analysis was performed on 5000 repetitions, and it was taken into account that the lower and upper limits in the 95% confidence interval did not include the zero value (Hayes, 2022). Standardized path coefficients (β) were interpreted for magnitude as well as significance. Coefficients below 0.10 were described as very small, values between 0.10 and 0.29 as small, values between 0.30 and 0.49 as moderate, and values of 0.50 and above as large (Cohen, 1988; Kline, 2015). Explained variance was additionally expressed as Cohen (1988) f^2^, computed as R^2^/(1 − R^2^), for which values of 0.02, 0.15 and 0.35 correspond to small, medium and large effects. These conventions are used descriptively; given the sample size, statistical significance alone was not treated as evidence of substantive importance. Finally, Cronbach’s alpha (α), CR, and AVE values were examined to evaluate the reliability and validity of the scales used in the study. Cronbach’s α and CR values above 0.70 and AVE values above 0.50 indicate that the scales are reliable and valid (Fornell and Larcker, 1981). Discriminant validity was assessed with the Fornell–Larcker criterion and the heterotrait–monotrait ratio of correlations (HTMT) (Henseler et al., 2015).

**TABLE 3:** Comparison of alternative measurement models.

Model	χ ^2^	df	χ ^2^/df	CFI	TLI	IFI	RMSEA	SRMR
M1: one factor (all items)	11555.57	377	30.65	0.53	0.49	0.53	0.158	0.151
M2: four factors (LMX unidimensional, CTI, LE, IP)	4062.39	371	10.95	0.84	0.83	0.84	0.092	0.055
M3: seven correlated first-order factors	1155.21	356	3.24	0.97	0.96	0.97	0.044	0.041
M4: second-order LMX + CTI, LE, IP (hypothesized)	1229.23	367	3.35	0.96	0.96	0.96	0.045	0.045

All models were estimated with maximum likelihood on the same 1185 cases and the same 29 items so that the models are directly comparable; the values for M4 are those obtained in this comparative run. M1–M3 are nested in or alternatives to M4. LMX, leader-member exchange; CTI, collective teacher innovativeness; LE, learning effectiveness; IP, instructional practices.

## 3 Findings

### 3.1 Descriptive statistics

Descriptive statistics of the study and Pearson correlation analysis results are given in Table 2.

As shown in Table 2, the arithmetic mean for the LMX variable (*M* = 3.35; SD = 0.92) indicates a medium level of perception. It is seen that the means for the variables CTI (*M* = 3.45; SD = 0.90), LE (*M* = 4.01; SD = 0.54), and IP (*M* = 3.83; SD = 0.60) are at a level that can be considered high. When SD values are examined, it is seen that the highest variance is in LMX (SD = 0.92) and CTI (SD = 0.90); the lowest variance is in LE (SD = 0.54). This result indicates that there are large differences among teachers in perceptions of LMX and CTI, while there is a more homogeneous distribution in LE. According to Pearson Correlation analysis, the LMX variable is positively and significantly correlated with CTI (*r* = 0.307, *p* < 0.01), LE (*r* = 0.262, *p* < 0.01), and IP (*r* = 0.242, *p* < 0.01). Similarly, the CTI variable shows significant positive relationships with both LE (*r* = 0.214, *p* < 0.01) and IP (*r* = 0.293, *p* < 0.01). A moderate positive correlation is also found between LE and IP (*r* = 0.430, *p* < 0.01). The pattern of correlations is consistent with the hypothesized model, although the bivariate coefficients are small to moderate in size and do not by themselves test the mediation structure.

### 3.2 Measurement model

Before proceeding to hypothesis testing, the measurement model in which all latent structures were estimated simultaneously was evaluated. The measurement model demonstrated an acceptable fit to the data (χ^2^/df = 3.35; RMSEA = 0.045; CFI = 0.96; TLI = 0.96; IFI = 0.96; GFI = 0.93; AGFI = 0.92; SRMR = 0.045). The standardized factor loadings for all structures and dimensions, along with Cronbach’s alpha (α), CR, and AVE values, are presented in Table 4.

**TABLE 4:** Standardized factor loading, reliability, and convergent validity values for the measurement model.

Dimension	Item	λ *	α	CR	AVE
Emotional interaction	L1	0.91	0.94	0.94	0.84
	L2	0.92			
	L3	0.92			
Contribution	L4	0.89	0.92	0.93	0.81
	L5	0.92			
	L6	0.89			
Loyalty	L7	0.74	0.87	0.88	0.71
	L8	0.87			
	L9	0.91			
Professional respect	L10	0.90	0.95	0.95	0.87
	L11	0.94			
	L12	0.96			
CTI	C1	0.83	0.92	0.93	0.77
	C2	0.92			
	C3	0.90			
	C4	0.85			
LE	LE1	0.45	0.75	0.80	0.42
	LE2	0.78			
	LE3	0.86			
	LE4	0.70			
	LE5	0.52			
	LE6	0.43			
IP	I1	0.68	0.83	0.86	0.48
	I2	0.90			
	I3	0.62			
	I4	0.58			
	I5	0.50			
	I6	0.74			
	I7	0.75			

*λ, standardized factor loading.

#### 3.2.1 Validity and reliability

As shown in Table 4, the convergent validity of the measurement model was assessed using standardized factor loadings, Cronbach’s alpha (α), CR, and AVE values. Cronbach’s alpha and CR values for all dimensions were found to be above the recommended threshold of 0.70, indicating that the scales possess adequate internal consistency. The AVE values met the recommended criterion of 0.50 for the dimensions of emotional interaction (0.84), contribution (0.81), loyalty (0.71), professional respect (0.87), and CTI (0.77), indicating that convergent validity was established. The AVE values for LE (0.42) and IP (0.48) fell below the 0.50 criterion (Bagozzi and Yi, 1988; Fornell and Larcker, 1981), which means that less than half of the variance in these indicators is accounted for by their latent construct. This should not be treated as unproblematic. The item-level loadings in Table 4 show that the shortfall is concentrated in a few items: LE1 (0.45), LE5 (0.52) and LE6 (0.43) for LE, and I5 (0.50) and I4 (0.58) for IP, whereas the remaining items load between 0.62 and 0.90. Removing LE1 and LE6 would raise the AVE of LE to approximately 0.53, and removing I5 would raise the AVE of IP to approximately 0.52. These items were nevertheless retained. Both instruments were used in their validated form, and shortening a validated scale on the basis of the present sample would risk capitalizing on sample-specific variation and narrowing the content coverage of the constructs (Little et al., 1999). As a robustness check, the structural model was re-estimated with LE1, LE6 and I5 excluded; every structural coefficient changed by 0.02 or less (LMX → CTI = 0.34, LMX → LE = 0.23, LMX → IP = 0.10, CTI → IP = 0.21, LE → IP = 0.40), and no conclusion was altered. Convergent validity for LE and IP is therefore described as partially rather than fully established, and the associations involving these two constructs should be read with this measurement limitation in view. Their CR values (0.80 and 0.86) indicate adequate composite reliability, but reliability does not compensate for the lower proportion of explained indicator variance. Finally, discriminant validity was assessed using the heterotrait-monotrait correlation ratio (HTMT) (Henseler et al., 2015) in addition to the Fornell-Larcker criterion. The results are summarized in Table 5, which reports CR and AVE alongside the Fornell–Larcker comparison and the HTMT values. The square root of the AVE of each construct exceeds its correlations with all other constructs; the smallest margin is for LE (√AVE = 0.65 against a latent correlation of 0.48 with IP). The HTMT values calculated from the item-level correlation matrix ranged from 0.25 (CTI–LE) to 0.55 (LE–IP) among the three first-order constructs, from 0.20 to 0.34 between the LMX dimensions and these constructs, and from 0.66 (loyalty–professional respect) to 0.84 (emotional interaction–professional respect) among the four LMX dimensions. All values are below the 0.85 threshold, indicating adequate discriminant validity. The comparatively high values among the LMX dimensions are expected, since these dimensions are indicators of a single second-order construct in the structural model.

**TABLE 5:** Discriminant validity of the measurement model: composite reliability, AVE, Fornell–Larcker criterion and HTMT ratios.

Construct	CR	AVE	1	2	3	4	5	6	7
1. Affect	0.94	0.84	*0.92*	0.83	0.70	0.84	0.33	0.27	0.26
2. Contribution	0.93	0.81	0.83	*0.90*	0.71	0.75	0.29	0.30	0.27
3. Loyalty	0.88	0.71	0.70	0.69	*0.84*	0.66	0.20	0.32	0.22
4. Professional respect	0.95	0.87	0.84	0.75	0.65	*0.93*	0.34	0.21	0.21
5. CTI	0.93	0.77	0.32	0.28	0.19	0.34	*0.88*	0.25	0.33
6. LE	0.80	0.42	0.22	0.26	0.28	0.17	0.18	*0.65*	0.55
7. IP	0.86	0.48	0.25	0.27	0.21	0.21	0.31	0.48	*0.69*

CR, composite reliability; AVE, Average Variance Extracted. Italic values on the diagonal are the square roots of AVE; values below the diagonal are latent correlations from the measurement model; values above the diagonal are HTMT ratios computed from the item-level correlation matrix. CTI, collective teacher innovativeness; LE, learning effectiveness; IP, instructional practices. Affect, contribution, loyalty and professional respect are the four dimensions of LMX.

To examine whether the hypothesized measurement structure is preferable to theoretically plausible alternatives, four measurement models were compared under the same maximum-likelihood specification (Table 3): a one-factor model in which all 29 items load on a single latent variable; a four-factor model in which LMX is treated as unidimensional alongside CTI, LE and IP; a seven-factor model with the four LMX dimensions, CTI, LE and IP as correlated first-order factors; and the hypothesized model in which the four LMX dimensions are indicators of a second-order LMX factor. The one-factor and four-factor models fitted poorly (CFI = 0.53 and 0.84, respectively), whereas the seven-factor and second-order models both fitted acceptably and differed only slightly, with the second-order model being more parsimonious (11 fewer parameters) at a negligible cost in fit. The very poor fit of the one-factor model also indicates that a single common factor, whether substantive or method-related, cannot account for the covariation among the items. The second-order specification was therefore retained for the structural model.

### 3.3 Structural model

All hypotheses were tested within a single structural model. As shown in Figure 2 and Table 6, the structural model fitted the data acceptably (χ^2^/df = 3.13; RMSEA = 0.042; CFI = 0.96; TLI = 0.95; IFI = 0.96; GFI = 0.93; AGFI = 0.92; SRMR = 0.047). Perceived collective teacher innovativeness was positively associated with IP (β = 0.21, SE = 0.031, 95% CI [0.147, 0.271], *p* < 0.001), a small coefficient by the conventions adopted here. LE was positively associated with IP (β = 0.41, SE = 0.034, 95% CI [0.345, 0.481], *p* < 0.001), a moderate coefficient and the largest in the model. LMX had a small direct association with IP (β = 0.10, SE = 0.033, 95% CI [0.031, 0.160], *p* = 0.004). LMX was moderately associated with perceived collective innovativeness (β = 0.34, SE = 0.033, 95% CI [0.274, 0.404], *p* < 0.001) and had a small association with LE (β = 0.25, SE = 0.035, 95% CI [0.182, 0.321], *p* < 0.001). The total association between LMX and IP, combining the direct path and the two indirect paths, was β = 0.27 (SE = 0.033, 95% CI [0.206, 0.334], *p* < 0.001). These results are consistent with H1 to H5. Of the control variables, gender (β = −0.04, SE = 0.031, 95% CI [−0.103, 0.022], *p* = 0.204) and educational level (β = 0.01, SE = 0.028, 95% CI [−0.049, 0.061], *p* = 0.838) were not associated with IP, whereas professional seniority (β = −0.10, SE = 0.029, 95% CI [−0.160, −0.046], *p* < 0.001) and length of service with the current principal (β = 0.07, SE = 0.029, 95% CI [0.011, 0.127], *p* = 0.015) had very small associations with IP. LMX accounted for 11.6% of the variance in perceived collective innovativeness (R^2^ = 0.116, *f*^2^ = 0.13) and 6.3% of the variance in LE (R^2^ = 0.063, *f*^2^ = 0.07); LMX, the two mediators and the control variables together accounted for 28.8% of the variance in IP (R^2^ = 0.288, *f*^2^ = 0.40). The first two values indicate that most of the variation in the mediators lies outside the model; the third is a substantial proportion by Cohen (1988) conventions, though it is shared among five predictors.

**FIGURE 2:**
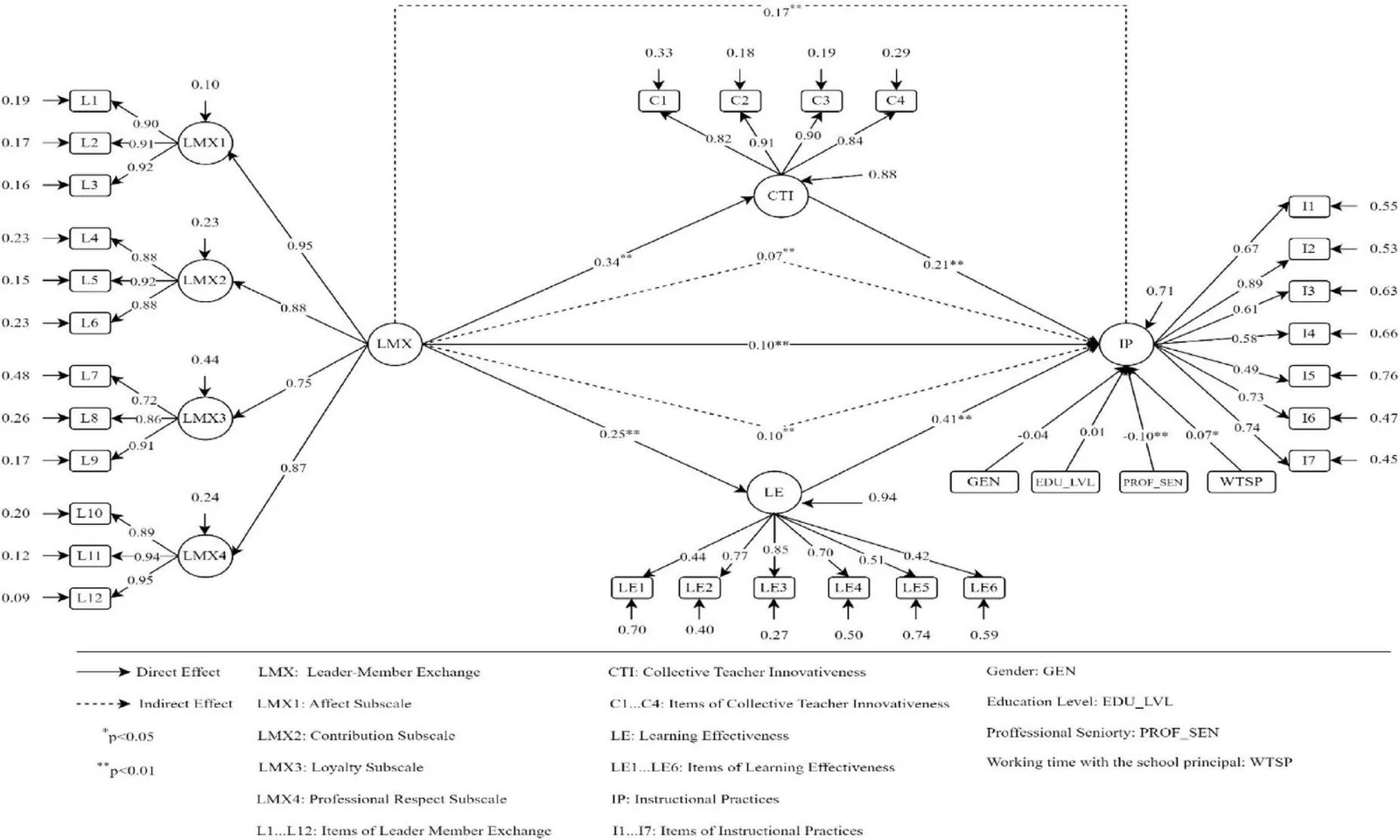
Analysis results regarding the structural relationships between the variables of the study.

**TABLE 6:** Bootstrapping analysis results for mediation models.

Construct	β	SE	95 % CI		R^2^	*p*
			LCI	UCI		
Standardized total effects						
LMX → IP	0.27^**^	0.033	0.206	0.334	0.288	0.000
Standardized direct effects						
CTI → IP	0.21^**^	0.031	0.147	0.271	–	0.000
LE → IP	0.41^**^	0.034	0.345	0.481	–	0.000
LMX → IP	0.10^**^	0.033	0.031	0.160	–	0.004
LMX → CTI	0.34^**^	0.033	0.274	0.404	0.116	0.000
LMX → LE	0.25^**^	0.035	0.182	0.321	0.063	0.000
Gender → IP	−0.04	0.031	−0.103	0.022	–	0.204
Education level → IP	0.01	0.028	−0.049	0.061	–	0.838
Professional seniority	−0.10^**^	0.029	−0.160	−0.046	–	0.000
Working time with the school principal → IP	0.07^*^	0.029	0.011	0.127	–	0.015
Standardized indirect effects						
LMX → CTI → IP	0.07^**^	0.013	0.048	0.100	–	0.000
LMX → LE → IP	0.10^**^	0.018	0.072	0.141	–	0.000
Standardized total indirect effect						
Total indirect effect (LMX → IP; via CTI and LE)	0.17^**^	0.022	0.136	0.221	–	0.000

*p < 0.05;

^**^p < 0.01; β, standardized beta coefficient; SE, standard error; CI, confidence interval; LCI, lower confidence interval; UCI, upper confidence interval; LMX, leader-member exchange; CTI, collective teacher innovativeness; LE, learning effectiveness; IP, instructional practices; Gender, the reference group is female; Education Level, the reference group is undergraduate. The total indirect effect is the sum of the two specific indirect effects; because the mediators are specified in parallel, it does not represent a separate pathway (see H6c). Exact *p*-values are reported; 0.000 denotes *p* < 0.001.

### 3.4 Bootstrap analysis

Bootstrap analysis was conducted to examine the indirect associations in the mediation model. The results of the bootstrap analysis are presented in Table 6.

Indirect associations were evaluated with 95% bootstrap confidence intervals based on 5000 resamples; an interval that excludes zero indicates a statistically significant indirect effect (Hayes, 2022; Preacher and Hayes, 2008). Table 6 distinguishes four quantities: the total association between LMX and IP, the direct association, the two specific indirect effects, and their sum. The specific indirect effect of LMX on IP through perceived collective innovativeness was β = 0.07 (SE = 0.013, 95% CI [0.048, 0.100], *p* < 0.001), and the specific indirect effect through LE was β = 0.10 (SE = 0.018, 95% CI [0.072, 0.141], *p* < 0.001). Neither interval includes zero, which is consistent with H6a and H6b; both coefficients are very small to small in magnitude. The total indirect effect, which equals the sum of the two specific effects because the mediators are specified in parallel, was β = 0.17 (SE = 0.022, 95% CI [0.136, 0.221], *p* < 0.001). This result confirms hypothesis H6c; it does not represent a mechanism separate from those examined in H6a and H6b. The indirect component accounts for approximately 63% of the total LMX–IP association (0.17/0.27) and the direct component for the remaining 37%. The learning route (0.10) is roughly one and a half times the size of the collective route (0.07); however, the confidence intervals of the two specific effects overlap, so this difference is descriptive and was not tested as a formal contrast.

## 4 Discussion

The first finding of the study showed that teachers’ perceived collective innovativeness is positively associated with their classroom IP. The coefficient is small by the convention adopted in this study rather than strong, so the association should be described as consistent and reliable rather than as large. In other words, where teachers perceive their colleagues as collectively engaged in developing new ideas in teaching and learning, they also report instructional practices of higher quality. Our finding is parallel to other study findings showing the positive association of teacher collaboration and innovative school climate with teachers’ in-class IP (Bellibaş et al., 2025). However, previous studies generally focused on individual, organizational, and environmental factors affecting teacher innovativeness (Hsieh et al., 2026). The present study adds the perceived collective dimension to that picture by examining it alongside individual learning effectiveness within a single model, which is an extension of existing work rather than an original theoretical contribution. Additionally, as is known, individual innovativeness may be limited due to the centralized structure and curriculum pressures (Kılınç et al., 2018). Our findings are consistent with the view that a collectively innovative climate, as teachers perceive it, accompanies better instructional practice, which gives some support to the development of a collaborative culture in schools; because the design is cross-sectional, this remains an association rather than a demonstrated effect of such a culture. As to why the association takes this form, the organizational-learning mechanism discussed earlier provides a plausible explanation. When teachers perceive their colleagues as collectively innovative, they may gain access to new instructional ideas and practices that have already been tried by others. This may make it easier and less risky for teachers to adapt their own established practices. At the same time, the modest size of the coefficient may reflect the limits imposed by the centrally determined curriculum and assessment framework, which may restrict the extent to which teachers can incorporate colleagues’ innovative ideas into their own instruction.

The second finding of the study revealed that teachers’ learning effectiveness is positively associated with their IP, and that this is the strongest association in the model. This result supports previous studies emphasizing that teachers’ active participation in professional learning is reflected in classroom practices (Liu et al., 2016). In Türkiye, teachers’ professional learning opportunities are often limited to standardized in-service training. Nonetheless, teachers who believe they can learn effectively appear to implement methods more effectively to improve their classroom practices. The third finding of the study shows that LMX is directly associated with IP, although this direct association is the smallest in the model and roughly 63% of the total LMX–IP association is carried indirectly through the two mediators. The relational quality of the principal-teacher relationship therefore appears to matter for instructional practice mainly through what it is associated with, rather than on its own. This result is in line with studies emphasizing that trust-based relationships with school leaders are important in instructional transformation (Graen and Uhl-Bien, 1995). In Türkiye, school principals are mostly busy with bureaucratic duties. Moreover, the third study finding suggests that relational interaction with teachers can be a critical area in paving the way for pedagogical innovativeness (Bellibaş, 2023). The difference between these two coefficients also provides support for the theoretical argument. LE has a stronger association with instructional practice, whereas the direct association between LMX and IP is weaker. This pattern is consistent with the two-theory model, which suggests that teachers’ own learning plays a more direct role in translating knowledge into changes in classroom practice, while the influence of LMX on instructional practice is more likely to operate through teachers’ learning.

The fourth finding of the study showed that LMX is positively associated with teachers’ perceived collective innovativeness, and this is the second largest coefficient in the model. Lin (2007) explained that willingness to share knowledge develops through relationships among organizational members, which in turn leads to the emergence of inspiring ideas and creative mental activities. Consistent with Buske (2018) definition of collective teacher innovativeness as combined efforts toward a shared vision, our finding suggests that school leadership may be associated with creative ideas and an innovative atmosphere by strengthening teacher interaction. In recent years, leadership approaches encouraging an innovative collaboration climate among teachers have begun to come to the fore in literature (Nguyen et al., 2021). This demonstrates the importance of collaborative interaction to generate innovative ideas. While active collaboration motivates teachers to go beyond their own experience and capacities and to benefit from the ideas of others, low levels of collaboration can discourage teachers from taking personal risks and lead to isolation (Goddard et al., 2007). A plausible reading of this pattern in the Türkiye setting is institutional rather than cultural. Where curricular and assessment decisions are taken centrally, the collegial group is one of the few arenas in which teachers can legitimately depart from routine, so the perceived openness of colleagues carries correspondingly more weight. This pattern is not tied to a collectivist national culture, since the design contains no measure of cultural orientation and no comparison system against which such a claim could be tested. The theoretical significance of this finding is that the exchange relationship appears to be associated not only with how teachers perceive themselves, but also with how they perceive their colleagues as a group. This pattern is consistent with SET, which suggests that when teachers receive voluntary support and effort from their colleagues, they may develop more positive perceptions of the collective. This aspect of the finding would not have been captured by a design focusing only on individual innovativeness.

The results for our fifth hypothesis showed that LMX is positively associated with teachers’ learning effectiveness. This finding is consistent with previous findings pointing to the importance of trust-based principal-teacher relationships that support teachers’ engagement in professional learning (Liu et al., 2016). Considering the obstacles created by the centralized structure in Türkiye, the quality of principal-teacher relationships can be considered a critical supporting element for teachers’ active participation in learning processes. The findings indicate that the association between LMX and IP is partly indirect, running through perceived collective teacher innovativeness (H6a) and LE (H6b). Where the relationship between principal and teacher is reported as strong, teachers also report a more innovative collegial climate and greater confidence in their own professional learning, and these in turn accompany higher reported quality of instructional practice. This finding supports other findings that leadership indirectly contributes to pedagogical practices by promoting innovative and learning-oriented cultures (Bellibaş et al., 2022; Zeng and Xu, 2020). Despite the structural constraints that teachers face in their innovativeness and learning processes, the mediating pattern observed here is worth noting in the Türkiye context, even though the indirect associations themselves are small in magnitude.

Taken together, the two indirect pathways constitute the central theoretical point of the study, although, as noted below, it is an extension of existing work rather than a new framework. The relationship between teachers and their principals appears to be associated with instructional practice mainly through two factors: a perceived climate that supports trying new approaches and a belief in the value of continued professional learning, rather than through a direct association with classroom practice. This provides a more specific explanation of how leadership is related to teaching than the general view that leadership matters for instructional practice. It is also the relationship that the parallel-mediator model was designed to examine. The two theories help explain these pathways in different ways: SET explains why the relationship with the principal is associated with these two factors, while OLT explains why these factors are more closely related to changes in classroom practice than the principal-teacher relationship itself.

Because all constructs were measured at the same time and from the same source, the findings are consistent with the proposed relationships but cannot confirm the direction of these relationships. Other explanations are also possible. For example, the relationship may work in the opposite direction: teachers who already use innovative practices and engage effectively in learning may receive more support from their principals. In this case, instructional practice may shape the principal-teacher relationship as much as the relationship shapes instructional practice. The observed associations may also be influenced by other factors. For instance, a generally positive school climate may lead teachers to evaluate their relationship with the principal, their colleagues’ openness to change, their own learning, and their IP more positively at the same time. In addition, teachers who see themselves as more effective may evaluate both their relationship with the principal and their own instructional practices more favorably, which could particularly strengthen the observed direct association between LMX and IP. The current research design cannot distinguish between these possible explanations and the proposed model. Therefore, the findings should be interpreted as associations rather than evidence of causal relationships. For this reason, the wording used throughout this section is deliberate: the findings are described as associations that “accompany” or are “related to” one another, and no statement should be read as a claim that LMX causes changes in innovativeness, learning or instruction. Cross-sectional estimates of indirect effects can differ considerably from those obtained with longitudinal data even when the underlying process is genuinely mediational (Maxwell and Cole, 2007), and the design employed here does not permit the direction of the relationships to be established (Spector, 2019).

The magnitude of the associations also requires attention beyond statistical significance. With 1,185 respondents, even modest coefficients can reach conventional significance levels, so statistical significance alone does not indicate practical importance. Based on the conventions adopted in this study, two paths fall within the moderate range, while none reaches the large range: the path from LE to IP and the path from LMX to perceived collective innovativeness. The other associations are small, including the paths from perceived collective innovativeness to instructional practice and from LMX to IP, as well as the total LMX–instructional practice association. The direct LMX–instructional practice association is also small, while the two specific indirect effects range from very small to small. A similar pattern is observed in the explained variance. LMX explains 11.6% of the variance in perceived collective innovativeness and 6.3% in LE, indicating that most of the variation in these mediators is explained by factors outside the model. The full model explains 28.8% of the variance in IP. These findings therefore represent modest rather than large effects and should be interpreted accordingly. Nevertheless, they remain meaningful for two reasons. First, instructional practice is influenced by many factors, including curriculum, assessment requirements, workload, subject area, and student characteristics. Thus, even a small contribution from a relational factor may be relevant within these structural constraints. Second, the mediating factors may be more amenable to intervention than these broader structural conditions. Schools and education systems can influence professional learning opportunities and the collegial conditions that support shared innovation, whereas the centralized curriculum is largely beyond the control of individual schools. Therefore, effects of this size support realistic and targeted expectations about the role of relational leadership, rather than suggesting that stronger principal-teacher relationships alone would substantially improve instructional quality. Two external reference points help to place these figures. In correlational research in organizational settings, coefficients around 0.10 fall in the lower third of published effect sizes and coefficients around 0.30 in the upper third (Bosco et al., 2015); the present path coefficients therefore span the ordinary range of effects in this literature rather than standing out from it. In education more specifically, Kraft (2020) has argued that effects that appear small by conventional benchmarks can still be consequential when they concern conditions that are low in cost and broad in reach, and the quality of principal–teacher relationships is such a condition. Even so, this argument supports realistic expectations about relational leadership; it does not support treating the present coefficients as evidence that changing exchange quality would produce a corresponding change in practice, since no such change was observed.

It is worth stating precisely what the present study adds and what it does not. The association between school leadership and teachers’ instructional practice is well established (Bellibaş et al., 2021a,b), and learning-related mediation of that association has been demonstrated previously (Bellibaş et al., 2022). The antecedents of collective teacher innovativeness have likewise been examined in large cross-national designs (Buyukgoze et al., 2024; Nguyen et al., 2021). What has not previously been reported is the joint estimation of a perceived-collective route and an individual-learning route within a single model with LMX as the predictor, together with their relative magnitudes. The specific empirical addition is the finding that the learning route is approximately one and a half times the size of the perceived-collective route and that the two together carry about 63% of the total association. This is an extension of existing work in a new configuration and in a centralized system.

### 4.1 Conclusion and implications

This study shows that LMX is associated with IP both directly and, to a greater extent, through teachers’ perceived collective innovativeness and their learning effectiveness. The findings suggest that relational aspects of school leadership are relevant to instructional quality in Türkiye’s centralized education system, while the magnitude of the associations observed is modest. According to the model presented within the framework of social exchange theory and organizational learning theory, high-quality principal-teacher relationships are accompanied by stronger innovative orientations and stronger beliefs about professional learning. Two kinds of implication follow, and they are best kept apart.

Theoretically, the study supports a division of labor between the two frameworks rather than an extension of either. Social exchange theory accounts for why teachers invest discretionary effort when the exchange with the principal is experienced as reciprocal; organizational learning theory accounts for how that effort is converted into changed classroom routines. The contribution is the specification and joint estimation of two parallel routes with different referents, one concerning what a teacher perceives colleagues to be doing and one concerning what a teacher believes about their own learning. It is not a new theory of school leadership, and it is not presented as one.

From a practical perspective, and given the modest effect sizes, the findings suggest several areas for attention. First, professional development for principals could place greater emphasis on building high-quality principal-teacher relationships as an important leadership skill. Second, education systems could provide greater structural support for school-based professional learning, particularly because the learning pathway showed the stronger association with instructional practice. Third, teacher evaluation frameworks could recognize collective as well as individual efforts to improve teaching. However, because the evidence is correlational and cross-sectional, these suggestions should be viewed as areas for policy consideration rather than as proven ways to improve instructional practice. Any interventions based on these findings would need to be evaluated separately. The findings may also be relevant to other centralized education systems, but whether they apply in those contexts remains an empirical question that cannot be answered by the present study.

### 4.2 Limitations of the study and suggestions for future research

The results of the study should be evaluated within the framework of certain contextual and measurement limitations. The study has a cross-sectional design, so the relationships between the variables can only be interpreted at the correlational level. The mediation model was specified on theoretical grounds, but the data cannot establish temporal order, and cross-sectional estimates of indirect effects may differ substantially from those that would be obtained with longitudinal data (Maxwell and Cole, 2007). For this reason, causal language has been avoided throughout the sections “3 Findings” and “4 Discussion,” and reverse or reciprocal relationships remain possible; longitudinal or experimental designs are needed to test the direction of these relationships. Since all of the scales were completed by the same teachers at the same time, common-source and common-method variance may have inflated the associations, and social desirability may also have affected responses. The diagnostics reported in the section “2.3 Data analysis” reduce but do not remove this concern. Future studies should collect data from different sources, for example principals’ ratings of exchange quality, colleagues’ ratings of innovativeness and observed classroom practice, or should separate the measurement of predictors and outcomes in time. Although the sample was stratified by the Türkiye Statistical Institute’s Level 1 regions and the regional distribution followed proportional targets, participation within schools was voluntary and no individual-level sampling frame covering all teachers in Türkiye was available, so the Equal Probability of Selection Method (EPSEM) principle could not be applied. Teachers who chose to respond may differ systematically from those who did not, for example in their relationships with principals or their engagement with professional learning. Proportional regional coverage should therefore not be read as evidence of representativeness in other respects, and generalization to the population of teachers in Türkiye should be made with caution. The 18 cases excluded as multivariate outliers were not retained in the data file, so the estimates could not be re-examined with these cases included. A check using stricter exclusion criteria produced the same pattern of results (see section “2.3 Data analysis”), but the influence of the excluded cases themselves remains unknown. Additionally, teachers working at different school levels (preschool, elementary school, middle school, and high school) were evaluated within a single analysis. Although the constructs examined in the study were treated as teacher characteristics independent of school level, no measurement invariance analyses were conducted to demonstrate that these constructs were measured in the same way across different school levels. In this regard, it is recommended that future studies test measurement invariance across school levels. A further limitation concerns the nested structure of the data. Teachers are clustered within schools, but school identifiers were not collected, so the analyses assume independent observations and cannot account for school-level dependence. Because both LMX and perceived collective innovativeness may partly reflect school-level processes, standard errors may be underestimated, and the individual- and school-level components of these constructs cannot be separated. Future studies should record school membership so that intraclass correlations can be estimated, cluster-robust standard errors can be used and, where the data justify it, multilevel measurement and structural models can be tested. 5-point Likert-type scales may not be sufficient to reflect fine distinctions; therefore, 7- or 9-point Likert scales may be preferred. Finally, mixed methods combining quantitative and qualitative data can contribute to a more holistic and in-depth understanding of the subject.

## Data Availability

The raw data supporting the conclusions of this article will be made available by the author, without undue reservation.
